# Supplementary material for: Pan-cancer copy number analysis identifies optimized size thresholds and co-occurrence models for individualized risk stratification
Source: Nat Commun. 2025 Jul 2;16:6024. doi: 10.1038/s41467-025-61063-y (PMC12222647; doi:10.1038/s41467-025-61063-y)
Supplement: Supplementary file 1 — Supplementary Information [file 41467_2025_61063_MOESM1_ESM.pdf]

Pan-cancer copy number analysis identifies optimized size thresholds and co-occurrence models for individualized risk stratification

Minh P. Nguyen[1,2,3], William C. Chen[1,2,3#], Kanish Mirchia[1,2,3], Abrar Choudhury[1,2,3], Naomi Zakimi[1,2,3], Vijay Nitturi[4], Tiemo J. Klisch[4], Stephen T. Magill[5], Calixto-Hope G. Lucas[6,7], Akash J. Patel[4], David R. Raleigh[1,2,3#]

[1]Department of Pathology, University of California San Francisco, San Francisco, CA, USA
[2]Department of Neurosurgery, University of California San Francisco, San Francisco, CA, USA
[3]Department of Radiation Oncology, University of California San Francisco, San Francisco, CA, USA
[4]Department of Neurosurgery, Baylor College of Medicine, Houston, TX, USA
[5]Department of Neurological Surgery, Northwestern University, Chicago, IL
[6]Department of Pathology, Johns Hopkins University, Baltimore, MD, USA
[7]Department of Neurosurgery, Johns Hopkins University, Baltimore, MD, USA

[#]Authors for correspondence: william.chen@ucsf.edu, david.raleigh@ucsf.edu

Supplementary Information

Supplementary Figures

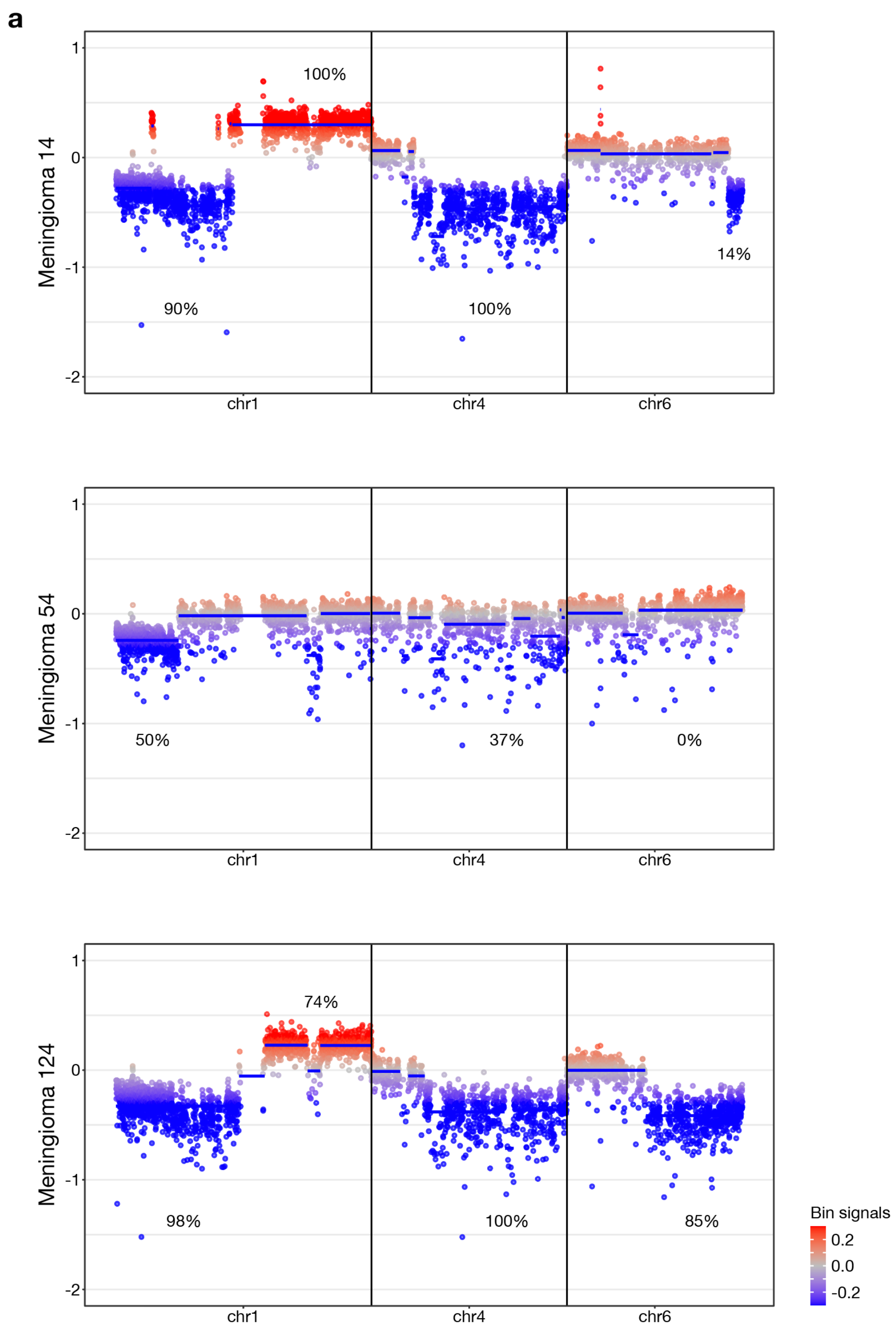

Supplementary Fig. 1. Meningioma CNA sizes are heterogenous across tumors. a, CNA profile plots showing segmentation data of select chromosome arms that are recurrently gained or lost in individual meningiomas. Thresholds for identifying/defining chromosome arm CNAs in individual meningiomas, below which a CNA would not be identified/defined, are also shown. The x-axis for each plot shows the entire length of the corresponding chromosome.

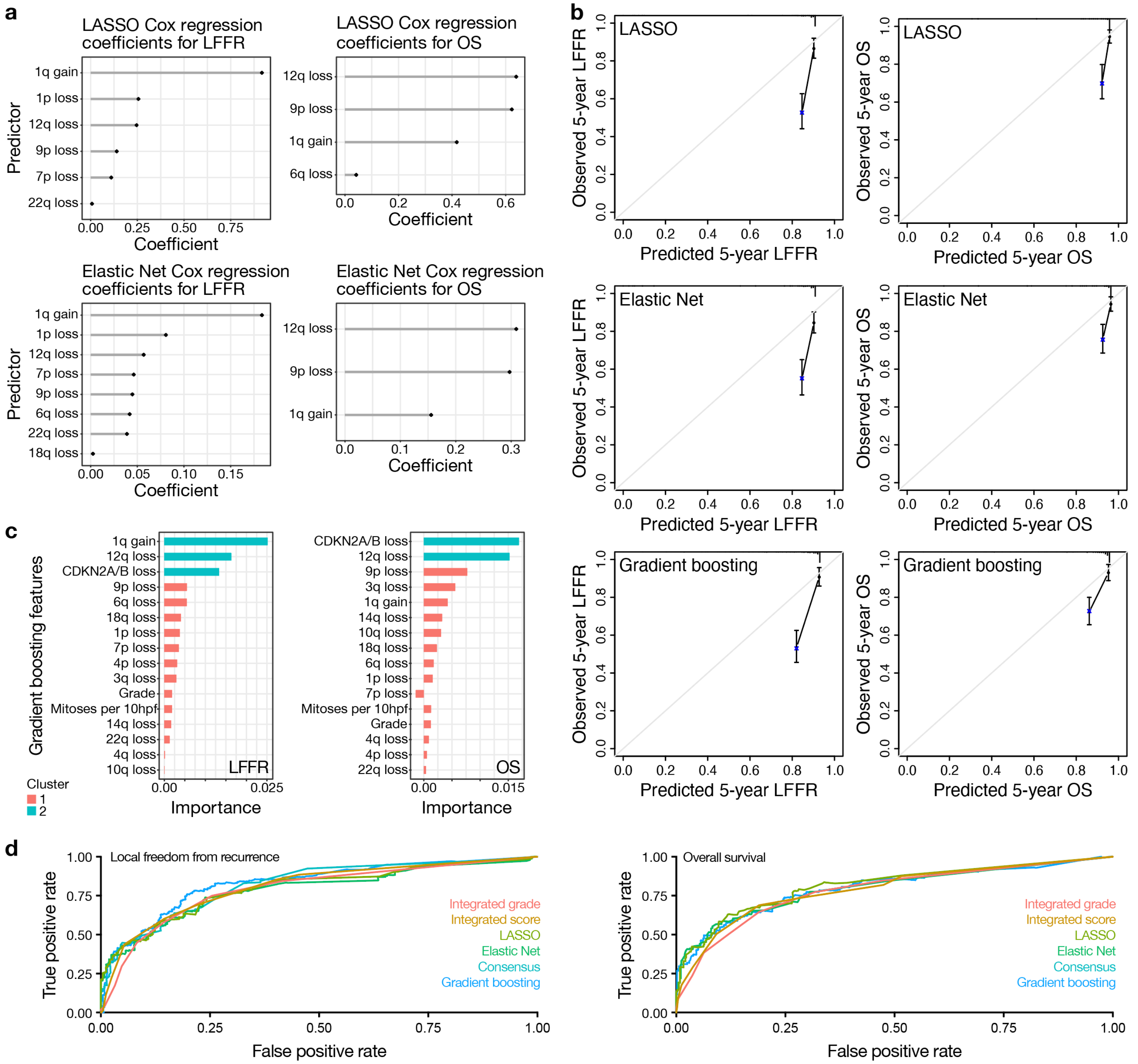

Supplementary Fig. 2. Size-dependent CNA models are prognostic and identify novel predictors of clinical outcomes. a, Coefficient plots for LASSO or Elastic Net regularized Cox models using LFFR or OS as outcomes and size-dependent CNAs as predictors. **b**, Calibration plots for LFFR (left) or OS (right) in the internal testing cohort for LASSO (top) and Elastic Net (middle) Cox models and gradient-booting (XGBoost) models (bottom). **c**, Variable importance plots from XGBoost models training to predict LFFR (left) or OS (right) using size-dependent CNAs, WHO 2016 histological grade, mitoses per 10 high-power field (HPF), and *CDKN2A/B* loss as predictors. **d**, Receiver-operating characteristic (ROC) curves for LFFR (left) and OS (right) in the internal testing cohort for size-dependent CNA LASSO and Elastic Net models, a Consensus model comprised of size-dependent CNAs that were shared across LASSO and Elastic Net models, and XGBoost models incorporating size-dependent CNAs, WHO 2016 histological grade, mitoses per 10 high-power field, and *CDKN2A/B* loss as predictors. In each panel, Integrated grade and Integrated score (with CNAs defined at the thresholds used to develop these models, 50% or 5%, respectively) are shown for comparison. Source data are provided as a Source Data file.

a

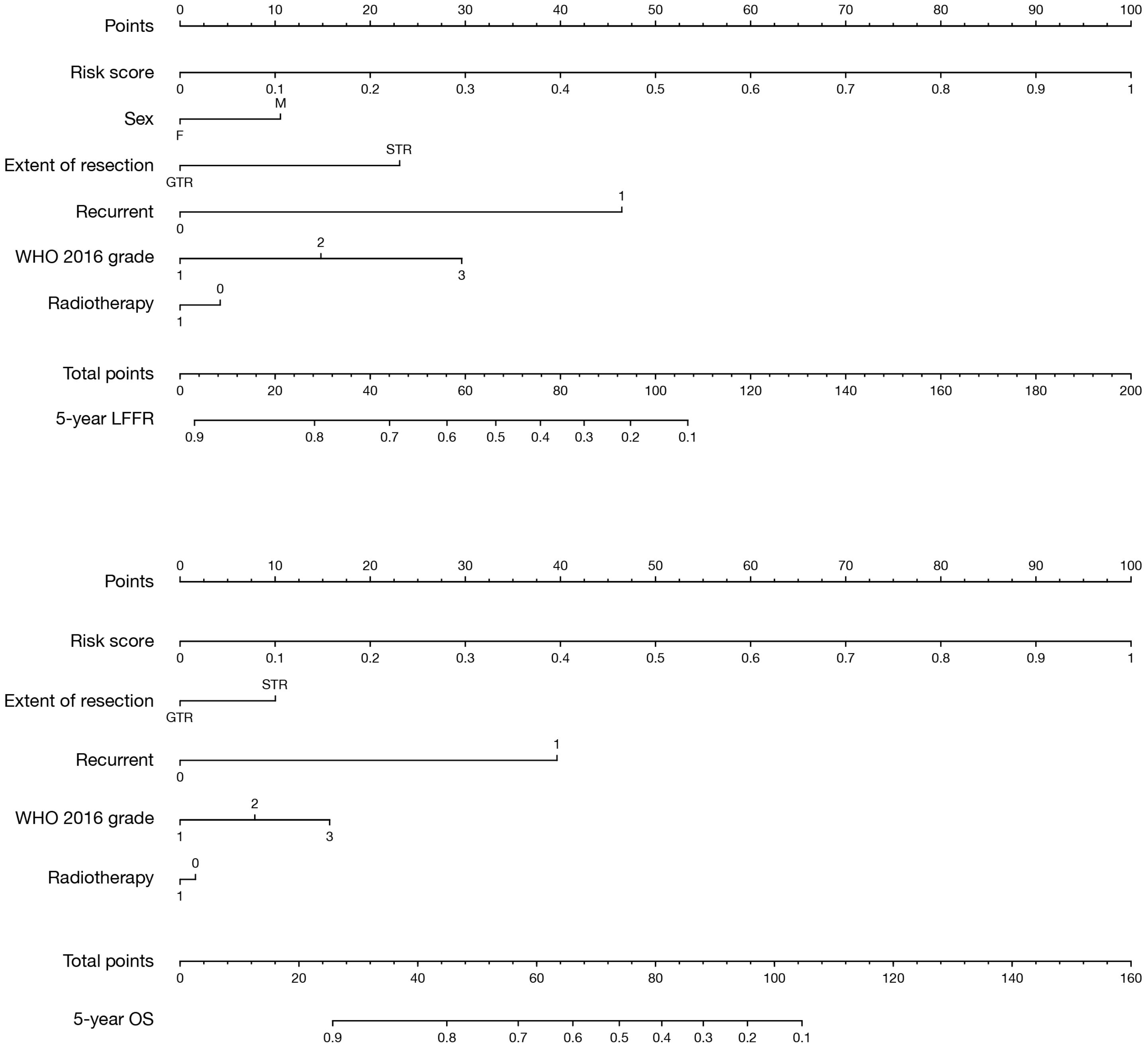

Supplementary Fig. 3. Nomograms for clinical implementation of size-dependent CNA models. a, Nomograms incorporating XGBoost models, sex, extent of resection (EOR), newly diagnosed versus recurrent presentation, and adjuvant radiotherapy to predict the risk of LFFR (top) or OS (bottom). To use the nomograms, use a straight-edge to draw a vertical line between the variable of interest and the points scale at the top of the nomogram to determine the contribution in points to the total score for each variable. Add up the points from each variable, and then draw a vertical line from the total points scale at the bottom of the nomogram to the 5-year outcome scale to determine the estimated outcome. The risk score variable is the output of the XGboost model risk prediction from panel Supplementary Fig. 2c linearly rescaled from 0 to 1.

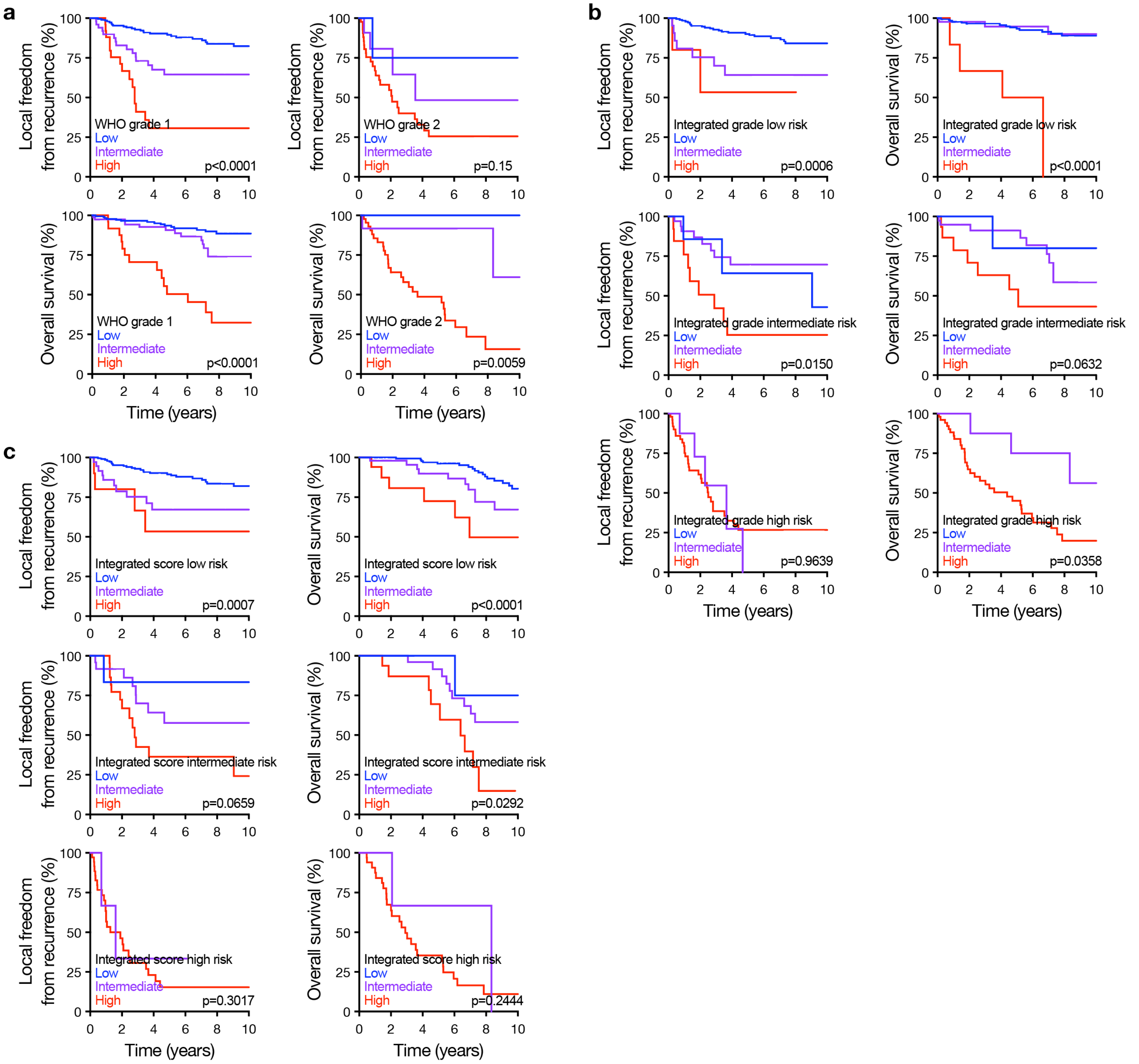

Supplementary Fig. 4. Size-dependent CNA models improve meningioma risk stratification within existing classification systems. a, Kaplan-Meier curves showing that XGBoost size-dependent CNA model strata (low/intermediate/high) identify higher risk meningiomas among WHO grade 1 (n=301) and WHO grade 2 (n=59) tumors, for both LFFR and OS. The number of WHO grade 3 meningiomas was too low for Kaplan-Meier analysis (n=5). **b**, Kaplan-Meier curves showing that XGBoost size-dependent CNA model strata (low/intermediate/high) identify higher risk meningiomas among low (n=244), intermediate (n=61), and high (n=60) risk tumors, as identified by Integrated grade, for both LFFR and OS. **c**, Kaplan-Meier curves showing that XGBoost size-dependent CNA model strata (low/intermediate/high) identify higher risk meningiomas among low (n=269) and intermediate (n=55) but not high (n=41) risk tumors, as identified by Integrated score, for both LFFR and OS. All data are from external test cohort 1, and all p-values are from Log-rank tests. Source data are provided as a Source Data file.

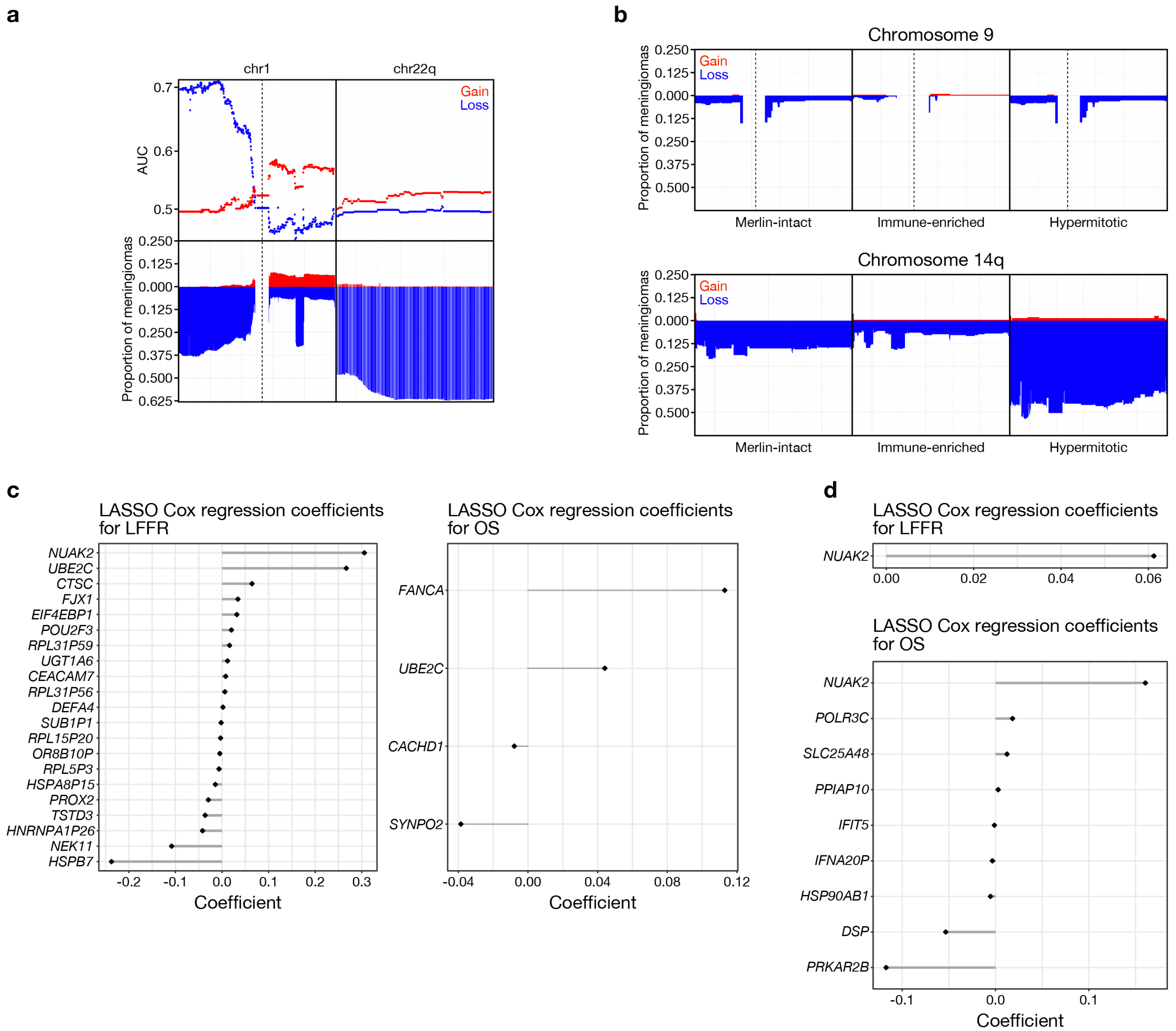

Supplementary Fig. 5. Focal CNAs in meningioma contain prognostic genes. a, CNA profile plots for chromosomes (bottom rows) demonstrating copy number losses (blue) or gains (red) in meningioma, and AUC plots (top rows) demonstrating prognostic significance associated with regions of loss (blue) or gain (red), in chromosomes 1p and 22q, two chromosomes without focal prognostic CNAs. Dashed line shows chromosome 1 centromere. The x-axis of each plot shows the entire length of the corresponding chromosome or chromosome arm. **b,** CNA profile plots for chromosomes 9 and 14 in meningiomas stratified by Merlin-intact, Immune-enriched, and Hypermitotic DNA methylation groups. Dashed lines show centromeres. The x-axis of each plot shows the entire length of the corresponding chromosome. **c**, Coefficient plots for LASSO models using LFFR or OS as outcomes and genes in focal regions of recurrent deletion across chromosomes in meningioma from Fig. 3a as predictors. **d**, Coefficient plots for LASSO models using LFFR or OS as outcomes and genes in focally prognostic regions across chromosomes in meningioma from Fig. 3a as predictors. Source data are provided as a Source Data file.

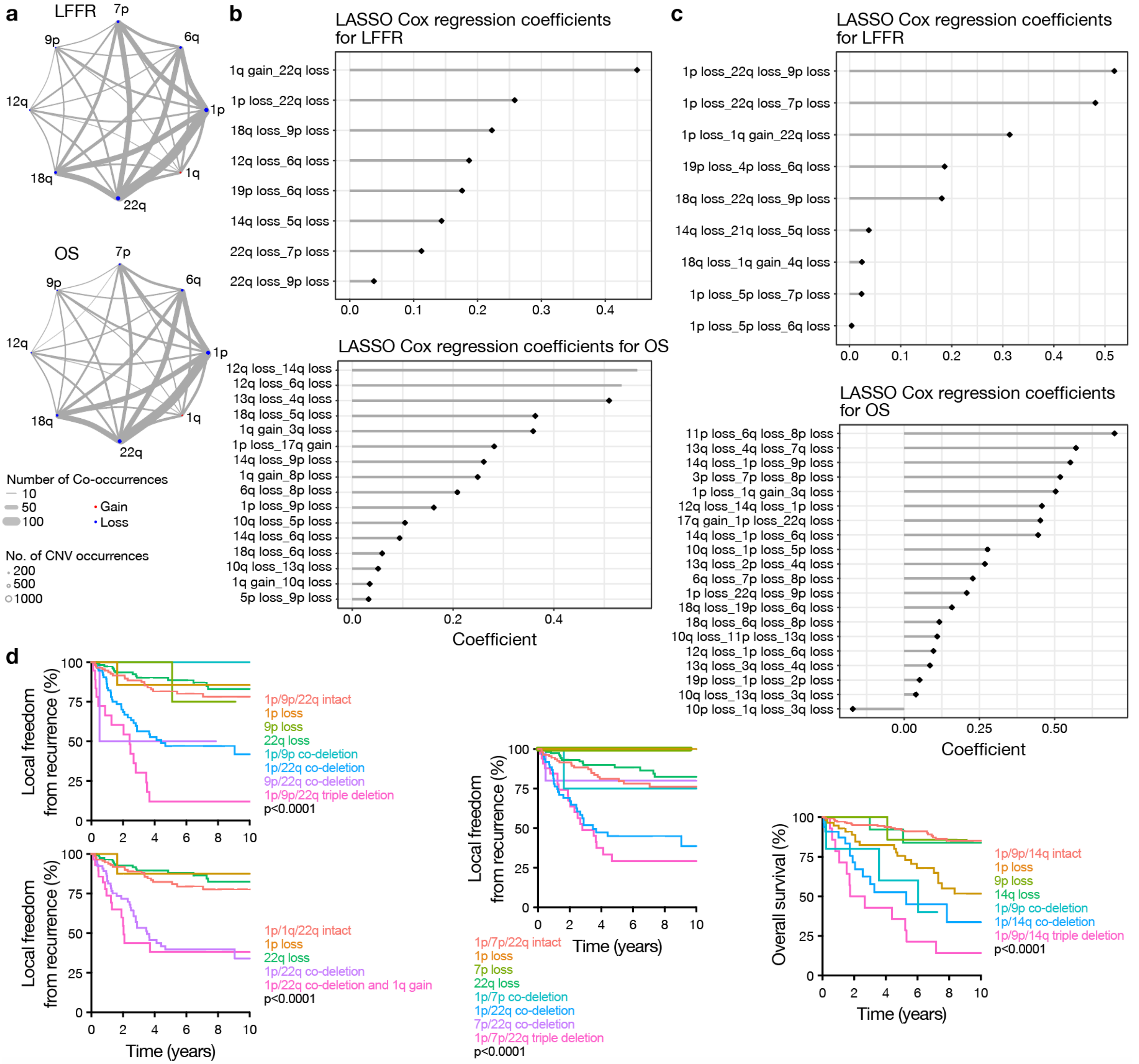

Supplementary Fig. 6. Co-occurrence modeling in meningioma identifies prognostic CNA pairs and triplets. a, Network diagrams demonstrating co-occurrence of prognostic size-dependent CNAs as defined using optimized thresholds for LFFR (top) or OS (bottom). Sizes of nodes correspond to the number of times a CNA co-occurred with another CNA in the network. Sizes of connecting edges corresponds to the number of times a connected pair co-occurred. **b**, Coefficient plots for LASSO Cox models using LFFR or OS as outcomes and co-occurring CNA pairs containing at least one size-dependent CNA as predictors. **c**, Coefficient plots for LASSO Cox models using LFFR or OS as outcomes and co-occurring CNA triplets containing at least one size-dependent CNA as predictors. **d**, Kaplan-Meier curves demonstrating risk stratification for LFFR and OS of CNA triplets from **c**. All p-values are from Log-rank tests (n=565 meningiomas). Source data are provided as a Source Data file.

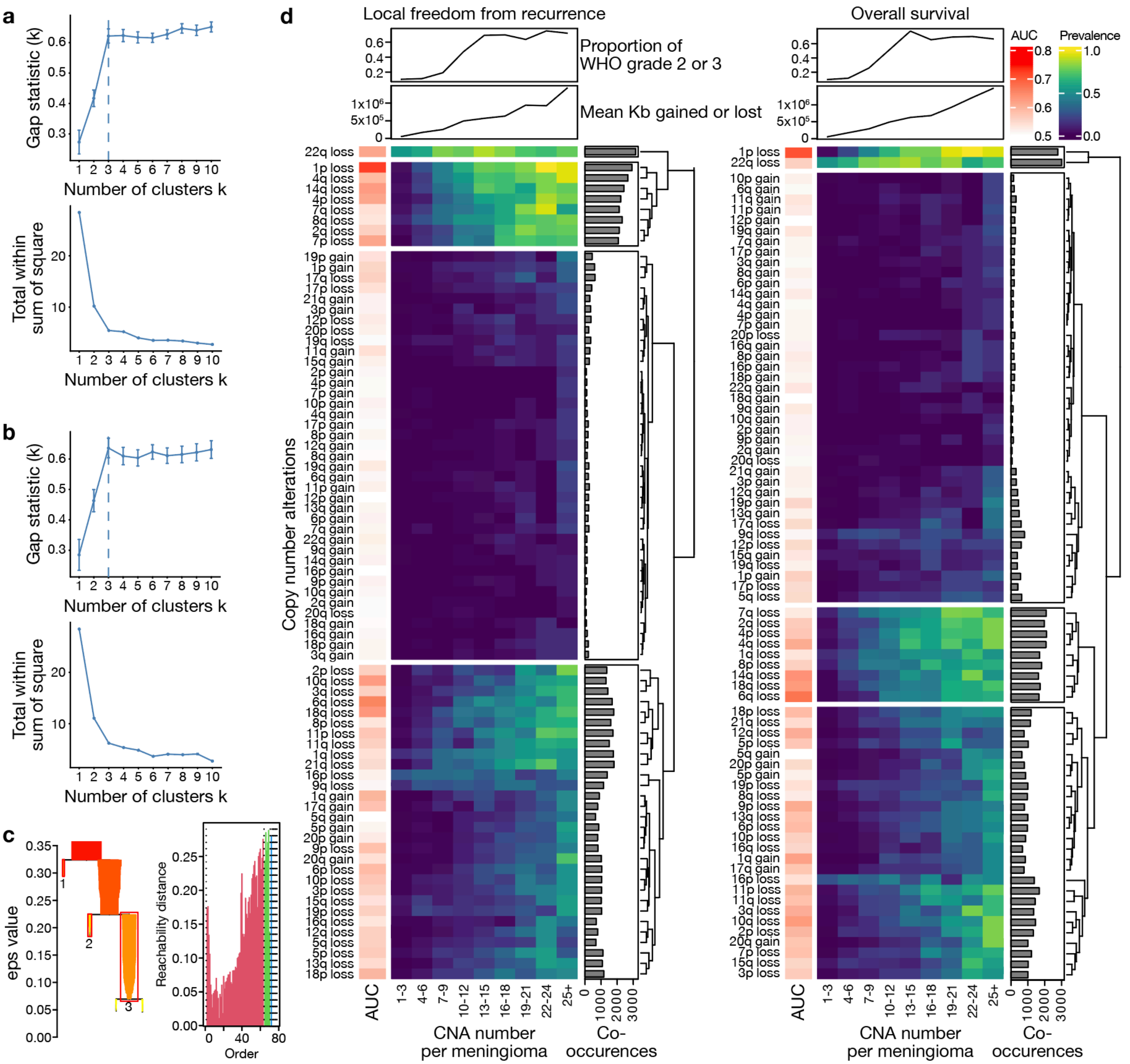

Supplementary Fig. 7. Calibration of CNA burden clustering. a, Elbow plot (top) and K-means plot (bottom) for clustering of CNAs called using optimal size thresholds for LFFR. **b**, Elbow plot (top) and K-means plot (bottom) for clustering of CNAs called using optimal size thresholds for OS. **c**, DBScan cluster tree plot (left) and reachability plot (right) identify 3 clusters of CNAs, like elbow and K-means plots. **d**, Heatmap showing unsupervised hierarchical clustering of individual CNAs according to the total number of CNAs per meningioma, with 4 clusters shown as a comparison to 3 clusters in Fig. 4b. CNAs were defined using optimal size thresholds for LFFR or OS. Bar plots on the right side of each heatmap measure the total number of co-occurrent pairs including each of the CNAs, across all samples containing that CNA.

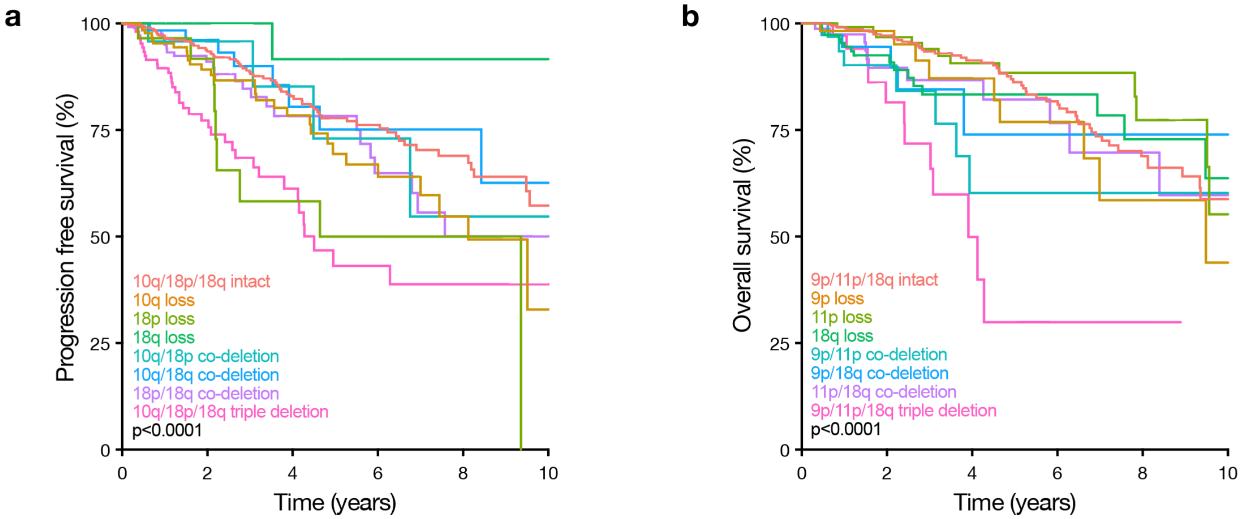

Supplementary Fig. 8. Co-occurrence CNA triplets are prognostic in breast cancer. a, Kaplan-Meier curve showing 10q/18p/18q co-deletion is associated with worse PFS for BCRA than any of these CNAs in isolation or in a pair, with the exception of 18p loss alone (n=1032). **b**, Kaplan-Meier curve showing 11p/18q/9p co-deletion is associated with worse OS in BRCA than any of these CNAs in isolation or in a pair (n=1032). All p-values are from Log-rank tests. These CNA triplets were selected for visualization based on LASSO Cox modeling of all co-occurring CNA triplets containing at least one size-dependent CNA. Source data are provided as a Source Data file.

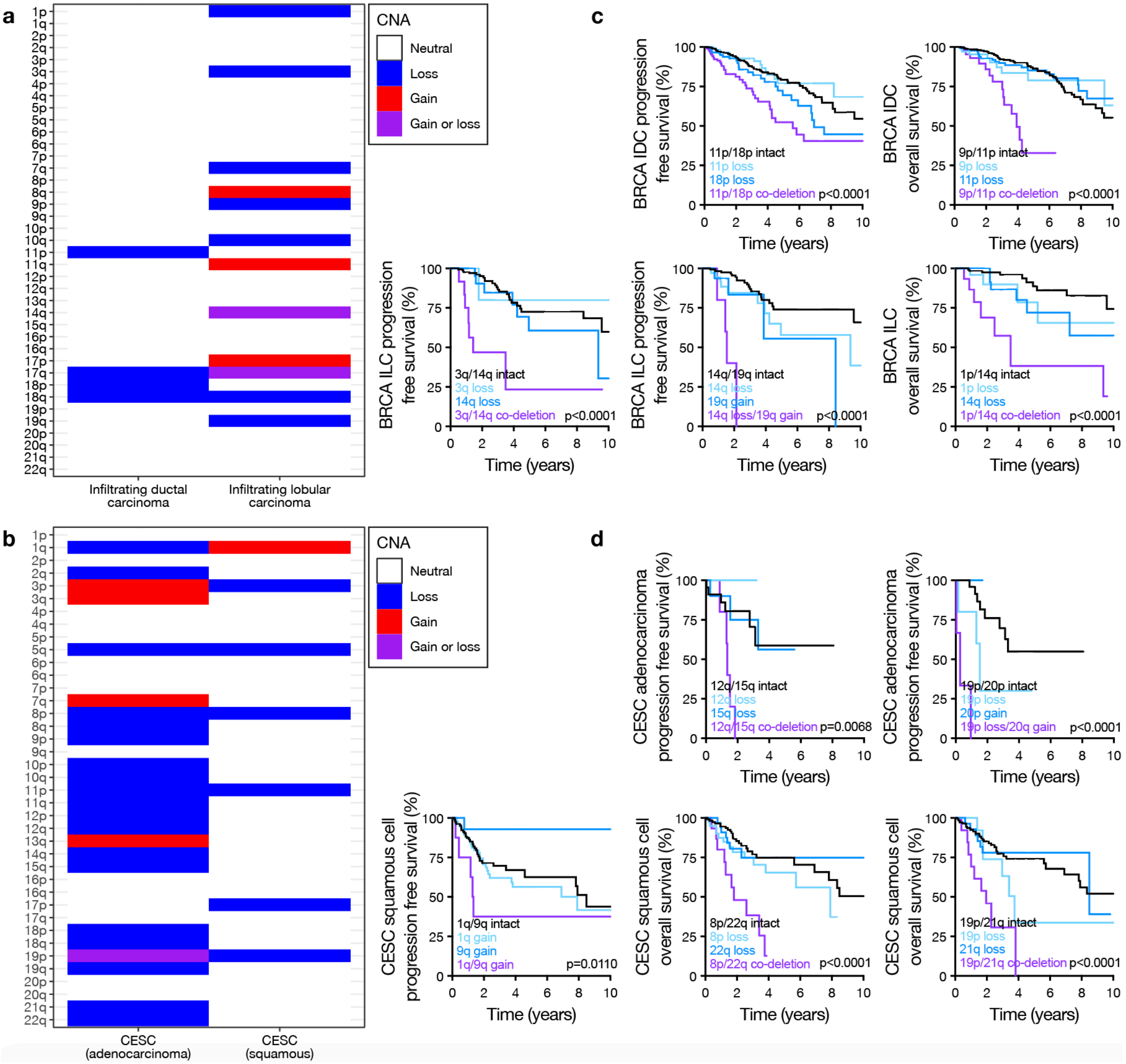

Supplementary Fig. 9. Histological subtypes of TCGA cancers have distinct size-dependent CNAs and CNA co-occurrence patterns that are associated with clinical outcomes. a, Heatmap demonstrating the presence of size-dependent CNAs in two histological subtypes of BRCA. **b**, Heatmap demonstrating the presence of size-dependent CNAs in two histological subtypes of CESC. Size-dependent CNAs were defined as having (1) a univariate Cox AUC for either PFS or OS of at least 0.60, (2) a standard deviation for AUC across CNA size thresholds of at least 0.01, and (3) presence in at least 2.5% of samples for a given TCGA cancer type. **c**, Kaplan-Meier curves showing prognostic co-occurring CNA pairs in infiltrating ductal BRCA (top, n=760) and infiltrating lobular BRCA (n=240, bottom). **d**, Kaplan-Meier curves showing prognostic co-occurring CNA pairs in CESC adenocarcinoma (n=43, top) and CESC squamous cell carcinoma (n=242, bottom). All p-values are from Log-rank tests. Kaplan-Meier curves show the most important co-occurrent pairs from LASSO Cox models for each cancer subtype. Source data are provided as a Source Data file.

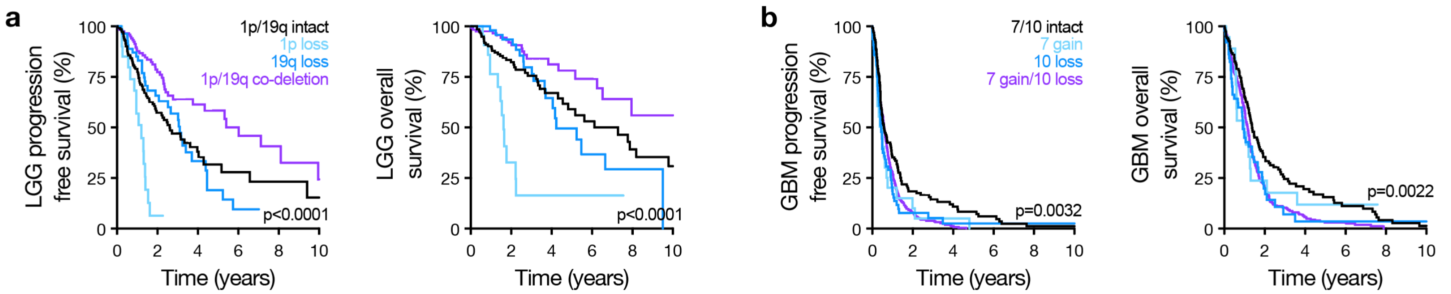

Supplementary Fig. 10. Select size-independent CNA co-occurrent pairs in TCGA. a, Kaplan-Meier curves demonstrate that 1p/19q co-deletion is associated with improved PFS and OS in LGG from TCGA (n=510). **b**, Kaplan-Meier curves demonstrate that concurrent loss of chromosome 7 and gain of chromosome 10 is not associated with any difference in OS or PFS compared to either of these CNAs alone in GBM from TCGA (n=571). All p-values are from Log-rank tests. Source data are provided as a Source Data file.
