# Supplementary material for: Pan-cancer copy number analysis identifies optimized size thresholds and co-occurrence models for individualized risk stratification
Source: Nat Commun. 2025 Jul 2;16:6024. doi: 10.1038/s41467-025-61063-y (PMC12222647; doi:10.1038/s41467-025-61063-y)
Supplement: Supplementary file 2 — Description of Additional Supplementary Files [file 41467_2025_61063_MOESM2_ESM.docx]

**Supplementary Data Legends**

**Supplementary Data 1. Size-dependence of meningioma CNAs.** Optimal size thresholds and 5-year area under the curve (AUC) for 5-year local freedom from recurrence (LFFR) or overall survival (OS) for each CNA. Standard deviations for each AUC and the proportion of samples across the integrated meningioma cohort (n=565) with each CNA called using the optimal thresholds were used to identify size-dependent CNAs.

**Supplementary Data 2. Percentage of meningiomas with CNAs across size thresholds.** Proportion of integrated meningioma cohort (n=565) with each CNA using increasing size thresholds. Optimal thresholds are those derived in Supplementary Data 1.

**Supplementary Data 3. Cox proportional hazards of size-dependent CNA models and clinical variables.** Multivariate Cox proportional hazards regressions to predict meningioma local freedom from recurrence (LFFR) or overall survival (OS) in External Test Cohort 1 (n=357) and External Test Cohort 2 (n=126). All p-values two-sided. No adjustments were made for multiple comparisons

**Supplementary Data 4. Focal recurrently gained or lost regions in meningioma.** Regions identified as focal recurrently gained or loss across chromosome arms as visualized in Fig. 3a, their start and end positions, and the genes located within them.

**Supplementary Data 5. Focally prognostic regions in meningioma.** Regions identified as focally prognostic for local freedom from recurrence (LFFR) across chromosome arms as visualized in Fig. 3a, their start and end positions, and the genes located within them.

**Supplementary Data 6. Multivariate Cox regression results of important co-occurrent pairs and triplets in meningioma.** Multivariate Cox proportional hazards regressions using co-occurrent CNA pairs and triplets to predict meningioma local freedom from recurrence (LFFR) (OS) in External Test Cohort 1 (n=365). All p-values two-sided. No adjustments were made for multiple comparisons.

**Supplementary Data 7. Size-dependent CNAs in TCGA.** Samples for each of the cancer types in the Pan-Cancer Atlas/The Cancer Genome Atlas (TCGA) included in analyses, their corresponding aneuploidy and copy number heterogeneity scores, and size-dependent CNAs.

**Supplementary Data 8. Optimal size thresholds and AUCs for size-dependent cancers in TCGA.** Optimal size thresholds and 5-year area under the curve (AUC) for 5-year progression-free survival (PFS) or overall survival (OS) for each CNA in TCGA cancer types.

**Supplementary Data 9. Prognostic co-occurring CNA combinations in TCGA.** Multivariate Cox proportional hazards regressions using co-occurrent CNA pairs to predict progression-free survival (PFS) and overall survival (OS) in size-dependent cancer types from TCGA. All p-values two-sided. No adjustments were made for multiple comparisons.

**Supplementary Data 10. Manual subtype assignment based on histological data in TCGA.** Cancer subtypes assigned based on available histological data in TCGA based on current WHO criteria.
