# Supplementary material for: Pan-cancer copy number analysis identifies optimized size thresholds and co-occurrence models for individualized risk stratification
Source: Nat Commun. 2025 Jul 2;16:6024. doi: 10.1038/s41467-025-61063-y (PMC12222647; doi:10.1038/s41467-025-61063-y)
Supplement: Supplementary file 4 — Reporting Summary [file 41467_2025_61063_MOESM4_ESM.pdf]

nature portfolio

Corresponding author(s): David R. Raleigh, MD, PhD

Last updated by author(s): Dec 26, 2024

Reporting Summary

Nature Portfolio wishes to improve the reproducibility of the work that we publish. This form provides structure for consistency and transparency in reporting. For further information on Nature Portfolio policies, see our Editorial Policies and the Editorial Policy Checklist.

Statistics

For all statistical analyses, confirm that the following items are present in the figure legend, table legend, main text, or Methods section.

n/a	Confirmed	
☐	☒	The exact sample size (*n*) for each experimental group/condition, given as a discrete number and unit of measurement
☐	☒	A statement on whether measurements were taken from distinct samples or whether the same sample was measured repeatedly
☐	☒	The statistical test(s) used AND whether they are one- or two-sided<br>*Only common tests should be described solely by name; describe more complex techniques in the Methods section.*
☐	☒	A description of all covariates tested
☐	☒	A description of any assumptions or corrections, such as tests of normality and adjustment for multiple comparisons
☐	☒	A full description of the statistical parameters including central tendency (e.g. means) or other basic estimates (e.g. regression coefficient) AND variation (e.g. standard deviation) or associated estimates of uncertainty (e.g. confidence intervals)
☐	☒	For null hypothesis testing, the test statistic (e.g. *F*, *t*, *r*) with confidence intervals, effect sizes, degrees of freedom and *P* value noted<br>*Give P values as exact values whenever suitable.*
☒	☐	For Bayesian analysis, information on the choice of priors and Markov chain Monte Carlo settings
☐	☒	For hierarchical and complex designs, identification of the appropriate level for tests and full reporting of outcomes
☒	☐	Estimates of effect sizes (e.g. Cohen's *d*, Pearson's *r*), indicating how they were calculated

Our web collection on statistics for biologists contains articles on many of the points above.

Software and code

Policy information about availability of computer code

Data collection	No software was used for data collection.
Data analysis	No custom software, tools, or packages were used. The open-source software, tools, and packages used for data analysis in this study are referenced in the methods where applicable and include R (v4.2.2), FASTQC (v0.11.9), HISAT2 (v2.1.0), Kallisto (v0.46.2), SeSAMe (v1.12.9) (BioConductor 3.13), survival R package (v3.5-7), survivalROC R package (v1.0.3.1), biomaRt R package (v2.54.1), glmnet R package (v4.1-8), igraph R package (v1.5.1), factoextra R package (v1.0.7), cluster R package (v2.1.4), ComplexHeatmap R package (v2.15.4), GSEA (v4.3.2), and EnrichmentMap App (v3.3.4) and AutoAnnotate app (v1.3.5) in Cytoscape (v3.7.2).

For manuscripts utilizing custom algorithms or software that are central to the research but not yet described in published literature, software must be made available to editors and reviewers. We strongly encourage code deposition in a community repository (e.g. GitHub). See the Nature Portfolio guidelines for submitting code & software for further information.

Data

Policy information about availability of data

All manuscripts must include a data availability statement. This statement should provide the following information, where applicable:

- Accession codes, unique identifiers, or web links for publicly available datasets
- A description of any restrictions on data availability
- For clinical datasets or third party data, please ensure that the statement adheres to our policy

DNA methylation and RNA sequencing for meningiomas in Discovery cohort 1 and External validation cohort 1 have been deposited in the NCBI Gene Expression under the accessions GSE183656 (https://www.ncbi.nlm.nih.gov/geo/query/acc.cgi?acc=GSE183656), GSE101638 (https://www.ncbi.nlm.nih.gov/geo/query/acc.cgi?acc=GSE101638), and GSE212666 (https://www.ncbi.nlm.nih.gov/geo/query/acc.cgi?acc=GSE212666). DNA methylation and RNA sequencing for meningiomas in External validation cohort 2 have been deposited under the accession number GSE189521 (https://www.ncbi.nlm.nih.gov/geo/query/acc.cgi?acc=GSE189521). The publicly available GRCh38 (hg38, https://www.ncbi.nlm.nih.gov/assembly/GCF_000001405.39/), and Kallisto index v10 (https://github.com/pachterlab/kallisto-transcriptome-indices/releases) datasets were used in this study. TCGA data was collected from the publicly available TCGA PanCanAtlas (https://gdc.cancer.gov/about-data/publications/pancanatlas). Copy number information was obtained using the Copy Number dataset (broad.mit.edu_PANCAN_Genome_Wide_SNP_6_whitelisted.seg). Clinical information was obtained from the TCGA-Clinical Data Resource (CDR) Outcome dataset (TCGA-CDR-SupplementalTableS1.xlsx). Source data are provided with this paper.

Human research participants

Policy information about studies involving human research participants and Sex and Gender in Research.

Reporting on sex and gender	The clinical samples used in this study were retrospective and nonrandomized with no intervention, and all samples were interrogated equally. Sex of participants was collected from the electronic health record, and was included where applicable in regression modeling as a covariate.
Population characteristics	The clinical samples used in this study were retrospective and nonrandomized with no intervention, and all samples were interrogated equally. Thus, controlling for covariates among clinical samples is not relevant.
Recruitment	As part of routine clinical practice at UCSF, all patients who were included in this study signed a waiver of informed consent to contribute deidentified data to research projects.
Ethics oversight	This study complied with all relevant ethical regulations and was approved by the UCSF Institutional Review Board (13-12587, 17-22324, 17-23196 and 18-24633).

Note that full information on the approval of the study protocol must also be provided in the manuscript.

Field-specific reporting

Please select the one below that is the best fit for your research. If you are not sure, read the appropriate sections before making your selection.

☒ Life sciences ☐ Behavioural & social sciences ☐ Ecological, evolutionary & environmental sciences

For a reference copy of the document with all sections, see nature.com/documents/nr-reporting-summary-flat.pdf

Life sciences study design

All studies must disclose on these points even when the disclosure is negative.

Sample size	No statistical methods were used to predetermine sample sizes, but sample sizes in this study are similar or larger to those reported in previous publications. Data distribution was assumed to be normal, but this was not formally tested.
Data exclusions	No clinical or molecular data points were excluded from the analyses.
Replication	No replicates were used.
Randomization	The clinical samples used in this study were retrospective and nonrandomized with no intervention, and all samples were interrogated equally. Thus, controlling for covariates among clinical samples is not relevant.
Blinding	Investigators were blinded to conditions during clinical data collection and analysis of mechanistic or functional studies.

Reporting for specific materials, systems and methods

We require information from authors about some types of materials, experimental systems and methods used in many studies. Here, indicate whether each material, system or method listed is relevant to your study. If you are not sure if a list item applies to your research, read the appropriate section before selecting a response.

Materials & experimental systems

n/a	Involved in the study
☒	☐ Antibodies
☒	☐ Eukaryotic cell lines
☒	☐ Palaeontology and archaeology
☒	☐ Animals and other organisms
☐	☒ Clinical data
☒	☐ Dual use research of concern

Methods

n/a	Involved in the study
☒	☐ ChIP-seq
☒	☐ Flow cytometry
☒	☐ MRI-based neuroimaging

Clinical data

Policy information about clinical studies

All manuscripts should comply with the ICMJE guidelines for publication of clinical research and a completed CONSORT checklist must be included with all submissions.

Clinical trial registration

This was a retrospective non-randomized study of human tumor samples with no intervention and is not eligible for clinical trial registration.

Study protocol

This was a retrospective non-randomized study of human tumor samples with no intervention and is not eligible for clinical trial registration.

Data collection

Meningioma samples were collected from three sites, UCSF, University of Hong Kong, and Baylor College of Medicine. Samples from the UCSF cohort (n=200) were selected from the UCSF Brain Tumor Center Biorepository and Pathology Core in 2017, and comprised all available WHO grade 2 and 3 meningioma frozen samples, WHO grade 1 frozen samples with clinical follow-up of greater than 10 years (n=40) or those with the longest available clinical follow-up less than 10 years (n=47). The electronic medical record was reviewed for all patients in late 2018, and paper charts were reviewed in early 2019 for patients treated before the advent of the electronic medical record. The University of Hong Kong cohort (n=365) comprised consecutive meningiomas from patients treated at University of Hong Kong from 2000 to 2019 with frozen tissue that was sufficient for DNA methylation. The medical record was reviewed for all patients in late 2019. The cohort comprised of meningioma samples with methylation profiling from Baylor College of Medicine included previously published (n=110) and unpublished (n=16) samples with clinical outcomes data, including patient age at surgery, sex, race, tumor size, tumor location, preoperative embolization, extent of resection, histologic grade, MIB-1 index, and presence of brain invasion. Diagnostic imaging was rereviewed to define tumor location, extent of resection, and presence/date of local recurrence. For all cohorts, meningioma recurrence was defined as new radiographic tumor on magnetic resonance imaging after gross total resection, or progression of residual meningioma on magnetic resonance imaging after subtotal resection.Pan-cancer clinical data was collected from the TCGA PanCanAtlas (https://gdc.cancer.gov/about-data/publications/pancanatlas), and clinical information was obtained from the TCGA-Clinical Data Resource (CDR) Outcome dataset (TCGA-CDR-SupplementalTableS1.xlsx).

Outcomes

Clinical outcomes relevant to meningioma biology include local recurrence as assessed from magnetic resonance imaging studies and patient death as assessed from the electronic medical record. Outcomes relevant to pan-cancer analysis include tumor progression and patient death as reported in The Cancer Genome Atlas database.

March 2021
